# Discrimination of sustained VT in structural heart disease using LGE-CMR and computational simulation: moving beyond LVEF

**DOI:** 10.3389/fcvm.2026.1870223

**Published:** 2026-07-07

**Authors:** Kun Zuo, Kuibao Li, Lucheng Xu, Yiming Wang, Shiwei Lu, Zhaokai Kong, Zhenyin Fu, Jianjun Zhang, Ruiqing Dong, Ling Xia, Dongdong Deng, Zheng Liu

**Affiliations:** 1 Heart Center & Beijing Key Laboratory of Hypertension, Beijing Chaoyang Hospital, Capital Medical University, Beijing, China; 2 School of Biomedical Engineering, Dalian University of Technology, Dalian, China; 3 College of Biomedical Engineering & Instrument Science, Zhejiang University, Hangzhou, China; 4 Department of Cardiology, Dushu Lake Hospital Affiliated to Soochow University, Suzhou, China

**Keywords:** cardiac magnetic resonance, computational simulation, risk stratification, structural heart disease, ventricular tachycardia

## Abstract

**Background:**

Sustained ventricular tachycardia (VT) is a major cause of sudden cardiac death in structural heart disease (SHD). Risk stratification based on left ventricular ejection fraction (LVEF) remains suboptimal. This study aimed to develop an exploratory model integrating late gadolinium enhancement cardiac magnetic resonance (LGE-CMR) scar characteristics with computational VT simulation to discriminate sustained VT, compared against LVEF.

**Methods:**

In this single-centre retrospective study, consecutive patients with suspected SHD who underwent LGE-CMR were enrolled. Patients with LGE-positive underwent quantitative scar analysis, personalized digital heart-twin reconstruction, and virtual electrophysiological simulation.

**Results:**

Among 82 patients (mean age 59.1 years; 82.9% male), 15 experienced sustained VT. Lower LVEF was associated with sustained VT (*P* = 0.008; C-statistic: 0.730), as was visual LGE positivity (*P* = 0.023; C-statistic: 0.634). Among LGE-positive patients, those with sustained VT had a larger core scar [10.0 [5.9, 14.8]% vs. 2.2 [1.1, 4.7]%; *P* < 0.001], larger grey zone [14.0 [10.9, 14.7]% vs. 5.7 [2.3, 9.2]%; *P* < 0.001], higher VT inducibility [50.0 [22.9, 66.9]% vs. 0 (0, 21.1)%; *P* < 0.001], and a greater number of inducible VT circuits [2 [2, 4] vs. 0 [0, 2]; *P* < 0.001]. An integrated LGE-VTsim index, derived from these four parameters, effectively discriminated sustained VT (C-statistic: 0.816) and remained independently associated with sustained VT after adjusting for LVEF (OR: 1.029; 95% CI: 1.008–1.051; *P* = 0.007).

**Conclusion:**

The LGE-VTsim index, integrating quantitative LGE-CMR scar assessment with computational simulation, represents a promising exploratory parameter in identifying patients at risk of sustained VT in SHD, beyond LVEF.

## 1 Introduction

Sustained ventricular tachycardia (VT) is a major cause of sudden cardiac death (SCD) in patients with structural heart disease (SHD). Contemporary pharmaceutical management has substantially reduced the incidence of SCD in patients with heart failure. However, a persistent residual risk remains despite guideline-directed medical therapy. Implantable cardioverter–defibrillator (ICD) implantation has therefore been associated with a further reduction in SCD risk, underscoring the continued necessity of ICD therapy and highlighting the urgent need for more refined risk stratification tools (1). Current guidelines primarily rely on a left ventricular ejection fraction (LVEF) ≤ 35% to guide primary prevention ICD therapy (2). Nevertheless, a substantial proportion of SCD events occur in patients with SHD and LVEF >35% (3, 4), underscoring the limitations of LVEF as a standalone risk marker.

Late gadolinium enhancement cardiac magnetic resonance (LGE-CMR) enables detailed characterisation of the arrhythmogenic substrate, including delineation of the infarct core and the border zone (grey zone) that are critical for re-entrant VT circuits (5–7) Although LGE-based scar quantification predicts arrhythmic risk in both ischaemic (ICM) and non-ischaemic cardiomyopathies (NICM) (8, 9), image-derived parameters alone may not fully capture the dynamic electrophysiological properties that are critical for VT inducibility and maintenance.

Recent advances in computational modelling have enabled the creation of patient-specific “digital twins” that integrate LGE-CMR–derived scar morphology with simulated electrophysiology to non-invasively assess VT inducibility (10–12). This approach seeks to capture the complex interplay between scar architecture and arrhythmia vulnerability.

In this study, we retrospectively analysed patients with SHD undergoing LGE-CMR to address four questions: 1) whether LVEF ≤35% represents the optimal cut-off for identifying sustained VT; 2) whether visual LGE assessment outperforms LVEF; 3) whether a model combining quantitative LGE characteristics with computational VT simulation surpasses LVEF alone; and 4) whether such a model can differentiate sustained from non-sustained ventricular arrhythmias (VAs). Our objective was to develop an integrated VT identification tool and compared with LVEF-based approaches.

## 2 Materials and methods

### 2.1 Study design and population

This single-centre retrospective study was conducted at Beijing Chaoyang Hospital and consecutively enrolled patients with SHD who underwent LGE-CMR between September 2024 and February 2025. The study protocol was approved by the Institutional Review Board of Beijing Chaoyang Hospital (2025-KE-921), and all participants provided informed consent.

Eligible patients met at least one of the following criteria: (1) LVEF <50%; (2) prior myocardial infarction, including ST-segment elevation myocardial infarction (STEMI) or non-ST-segment elevation myocardial infarction (NSTEMI); (3) diagnosed cardiomyopathy, including dilated cardiomyopathy (DCM) or hypertrophic cardiomyopathy (HCM); (4) echocardiographic evidence of regional wall motion abnormalities; (5) electrocardiographic documentation of VA; or (6) planned implantation of an ICD or cardiac resynchronisation therapy defibrillator. Exclusion criteria were: (1) arrhythmogenic right ventricular cardiomyopathy; (2) inherited channelopathies; (3) age <18 years; (4) estimated glomerular filtration rate <30 mL/min; (5) contraindications to CMR; or (6) poor LGE-CMR image quality. Genetic cardiomyopathies were not systematically assessed owing to limited availability of genetic testing.

### 2.2 Clinical data collection

SHD was classified as ICM or NICM. ICM was diagnosed based on any of the following: a history of ischaemic heart disease or coronary revascularisation; coronary artery stenosis ≥50%; inducible ischaemia; or an LGE pattern consistent with myocardial infarction (13). NICM was defined by a non-coronary LGE distribution in the absence of significant coronary artery stenosis (14, 15). Data on LVEF, New York Heart Association (NYHA) functional class categories, comorbidities, and medications were systematically collected.

All patients underwent clinical and arrhythmia assessments within a 6-month peri-CMR period, including outpatient consultations, electrocardiography, ICD interrogations, and 24-hour Holter monitoring. Ventricular arrhythmias (VAs) were categorised as: (1) sustained VT (>30 s or requiring intervention due to haemodynamic compromise, including appropriate ICD therapy); (2) non-sustained VA [frequent premature ventricular contractions (PVCs) ≥ 1,000 per 24 h, with or without non-sustained VT] (16, 17); or (3) no clinically relevant VA (<1,000 PVCs per 24 h without sustained VT).

### 2.3 Study group definitions

Patients were classified into Event and Event-free groups according to the occurrence of sustained VT during the period from one week before to 6 months after the LGE-CMR examination. The Event group included patients with appropriate ICD therapy, SCD, or documented sustained VT, whereas all remaining patients comprised the Event-free group.

### 2.4 Image acquisition, digital twin reconstruction, and electrophysiological simulation

All participants underwent LGE-CMR using 3.0-T scanners (Siemens Prisma or Vida). Epicardial and endocardial contours were automatically segmented and manually refined to exclude the blood pool and epicardial fat. Myocardial tissue was then classified into non-scar and scar regions, with scar further subdivided into core scar (pixels >50% of the signal intensity range) and grey zone according to pixel intensity thresholds. Segmented images were interpolated to a 0.4 mm resolution using CardioViz3D. Scar geometry was reconstructed using the log-odds method and integrated into patient-specific biventricular finite-element meshes (approximately 400 μm element size), in which myocardial fibre orientation was assigned from −40° at the epicardium to +65° at the endocardium using a rule-based approach.

The electrophysiological properties of non-scared myocardium, grey zone, and core scar were simulated using the ten Tusscher model. Ionic current conductances in the grey zone were scaled to 30% (IKr), 20% (IKs), 31% (ICaL), and 38% (INa) of normal values, resulting in conduction velocities of 0.60 m/s in healthy myocardium and 0.30 m/s in the grey zone, while the core scar was treated as electrically passive. VT inducibility was assessed in the openCARP environment by applying a standardised rapid pacing protocol, consisting of a six-beat S1 train (600 ms) followed by up to three premature stimuli (S2–S4), at eight ventricular sites defined by the American Heart Association segmented model (18). Induced re-entrant VT was simulated for 10 s to confirm stability, and circuit locations were mapped for subsequent analysis. The LGE segmentation and virtual electrophysiological study pipeline was implemented in a largely automated manner to minimise operator-dependent variability. Throughout the entire process, the engineer performing the simulations was blinded to all patient outcomes to prevent bias.

### 2.5 Statistical analysis

Continuous variables are presented as mean ± standard deviation or median with interquartile range, and categorical variables as frequencies and percentages. Between-group comparisons were performed using the Chi-square test (*χ*^2^ test) or Fisher's exact test, as appropriate. Correlation analyses were performed to assess collinearity and variable interdependence, followed by exploratory factor analysis (EFA) to extract latent factors and reduce data dimensionality for subsequent modeling (LGE-VTsim index). Prior to EFA, all variables were standardized to z-scores (mean = 0, SD = 1) to eliminate scale differences and ensure equal contribution to the extracted factor. The discriminatory performance of LVEF, visual LGE assessment, and the LGE-VTsim index for sustained VT was evaluated using the C-statistic, with comparisons conducted using DeLong's test. Optimal cut-off values were determined by maximising the Youden index. Logistic regression was used to assess associations with outcomes, which are expressed as odds ratios (OR) with 95% confidence intervals (CI). The incremental discriminative value of the LGE-VTsim index over LVEF was assessed using the net reclassification index (NRI), integrated discrimination improvement (IDI), and decision-curve analysis (DCA). To address the risk of overfitting, optimism-corrected C-statistics were calculated via bootstrap resampling. Calibration of the model incorporating the LGE-VTsim index was assessed by comparing predicted probabilities with observed outcomes using a calibration plot with loess smoothing; the 45° line represented perfect calibration. Statistical analyses were performed using SPSS (version 31.0) and R (version 4.4.2). A two-sided *P* < 0.05 was considered statistically significant.

## 3 Results

### 3.1 Baseline characteristics and endpoints

**Figure 1:**
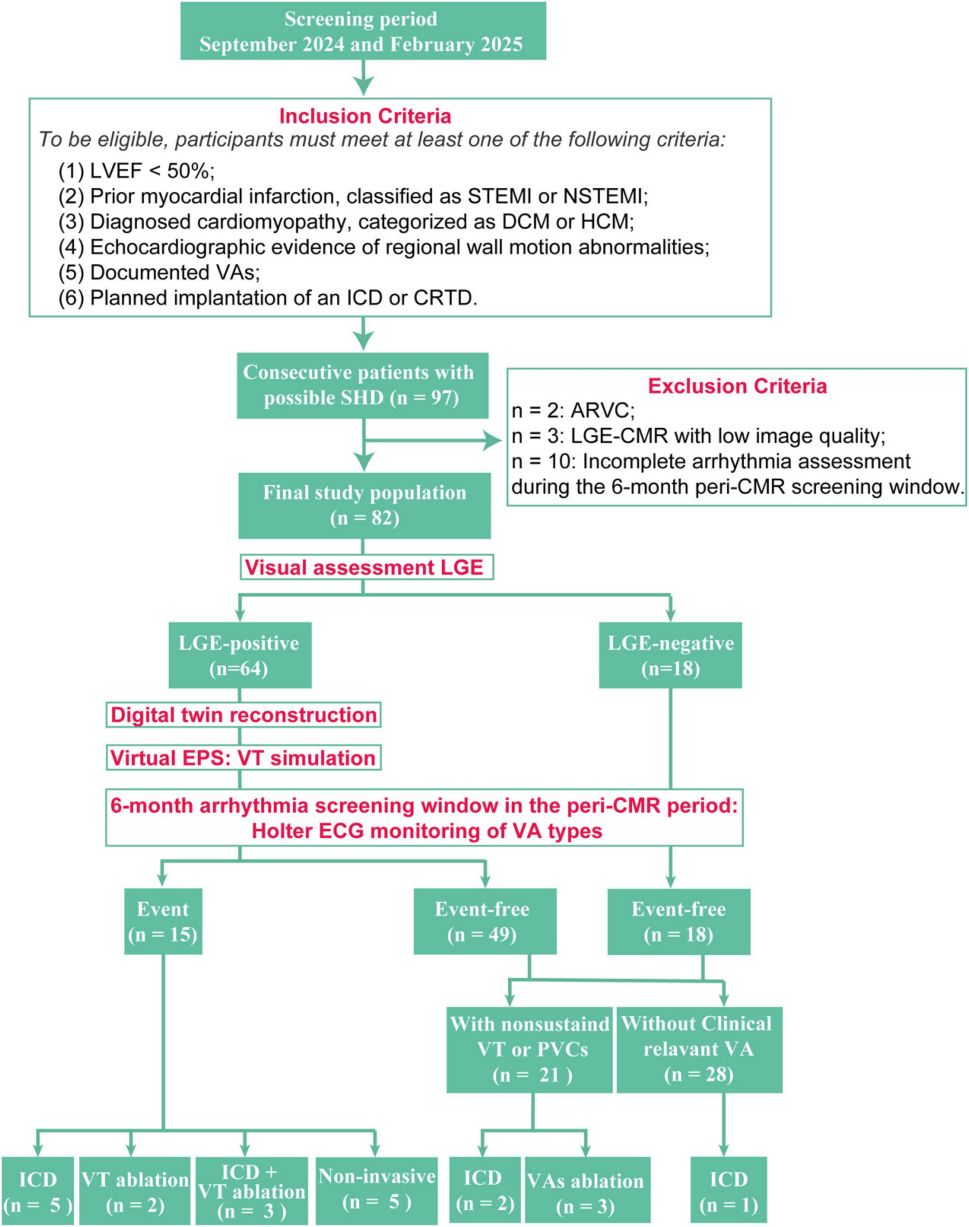
Study flow chart and clinical outcomes. The flowchart depicts the selection of patients with suspected SHD based on LGE-CMR findings and their subsequent classification by 6-month arrhythmic outcomes. LGE-CMR, late gadolinium enhancement cardiac magnetic resonance; VT, ventricular tachycardia; SCD, sudden cardiac death; ICD, implantable cardioverter-defibrillator; VA, ventricular arrhythmia; PVCs, premature ventricular contractions.

A total of 97 patients undergoing LGE-CMR were retrospectively enrolled. After exclusion of 15 patients (2 with arrhythmogenic right ventricular cardiomyopathy, 3 due to poor LGE-CMR image quality, and 10 with incomplete arrhythmia assessment during the 6-month peri-CMR screening window), 82 patients constituted the final study population (Figure 1). The mean age was 62.0 (53.0, 68.0) years, and the majority of patients were male (82.9%). The mean LVEF was 45.4% ± 14.4%; 20 patients (24.4%) had an LVEF ≤35%, and 64 (78.0%) were visually assessed as LGE-positive. Detailed clinical characteristics are presented in Table 1.

**Table 1:** Baseline characteristics of the study population.

Characteristic	Event (*n* = 15)	Event-free (*n* = 67)	P
Female	1 (6.7%)	13 (19.4%)	0.421
Age (years)	56 (53.5, 68.5)	62 (54, 67)	0.723
Body surface area (m^2^)	1.91 ± 0.12	1.86 ± 0.18	0.370
Ventricular arrhythmia Burden %	0.3 (0.2, 0.95)	0.3 (0.1, 1.15)	0.516
Diabetes	4 (30.8%)	20 (29.9%)	0.947
Hypertension	5 (38.5%)	37 (55.2%)	0.268
Ischemic cardiomyopathy	9 (60.0%)	44 (65.7%)	0.678
NYHA function class			
I	1 (6.7%)	14 (20.9%)	0.425
II	8 (53.3%)	35 (52.2%)	
III	5 (33.3%)	16 (23.9%)	
IV	1 (6.7%)	2 (3.0%)	
Medications			
*β*-blocker	15 (100%)	51 (76.1%)	0.035
ACEI/ARB	15 (100%)	49 (73.1%)	0.023
Aldosterone antagonist	14 (93.3%)	24 (35.8%)	<0.001
Loop diuretic	5 (33.3%)	11 (16.4%)	0.135
LGE-CMR			
LVEF %	35.6 ± 13.3	47.4 ± 13.9	0.008
LVEF ≤35%	7 (50.0%)	13 (19.4%)	0.016
LVEF>50%	1 (7.1%)	30 (44.8%)	0.008
LV mass (g)	153.8 (128.6, 174.1)	140.2 (105.0, 171.4)	0.450
RVEF (%)	50.3 ± 16.7	52.9 ± 10.3	0.534
LGE-positive	15 (100%)	49 (73.1%)	0.023

ACEI, angiotensin-converting enzyme inhibitor; ARB, angiotensin II receptor blocker; LV, left ventricular; LVEF, left ventricular ejection fraction; LGE, late gadolinium enhancement; NYHA, New York Heart Association; RVEF, right ventricular ejection fraction.

Fifteen patients experienced clinical sustained VT events (Event Group). Of these, 12 had VT episodes shortly before CMR, and 3 developed newly documented sustained VT after CMR, all of whom received appropriate ICD therapy. All patients in the Event Group were receiving *β*-blocker therapy. For clinical management, 8 patients received an ICD, 5 underwent VT ablation (including 3 with concurrent ICD implantation), and 5 declined invasive intervention (Figure 1).

### 3.2 Scar quantification and computational VT simulation in LGE-positive patients

Analyses focused on the 64 LGE-positive patients [age 62.0 (53.5, 69.0) years; 84.4% male], including 44 with ICM (30 with prior STEMI) and 20 with NICM (11 dilated, 7 hypertrophic, and 2 other types). The mean LVEF was 44.9% ± 15.2%, with 17 patients (26.6%) having an LVEF ≤35%.

Quantitative LGE analysis demonstrated that dense scar and grey zone occupied 3.6 (1.5, 7.8)% and 7.3 (2.8, 13.7)% of left ventricular myocardial tissue, respectively. Detailed characteristics of the LGE-positive patients are summarised in Table 2.

**Table 2:** Baseline characteristics of the LGE-positive patients.

Characteristic	Event in LGE-positive (*n* = 15)	Event-free in LGE-positive (*n* = 49)	P
Female	1 (6.7%)	9 (18.4%)	0.275
Age (years)	56.0 (53.5,68.5)	62.0 (55.0,69.0)	0.461
Body surface area (m^2^)	1.91 ± 0.12	1.87 ± 0.15	0.374
Ventricular arrhythmia Burden %	0.3 (0.2, 0.95)	0.3 (0.2, 2.5)	0.904
Diabetes	4 (30.8%)	14 (28.6%)	0.877
Hypertension	5 (38.5%)	24 (49.0%)	0.499
Ischemic cardiomyopathy	9 (60.0%)	35 (71.4%)	0.403
NYHA function class			
I	1 (6.7%)	12 (24.5%)	0.130
II	8 (53.3%)	27 (55.1%)	
III	5 (33.3%)	10 (20.4%)	
IV	1 (6.7%)	0 (0%)	
Medications			
β-blocker	15 (100%)	38 (77.6%)	0.044
ACEI/ARB	15 (100%)	37 (75.5%)	0.033
Aldosterone antagonist	14 (93.3%)	18 (36.7%)	<0.001
Loop diuretic	5 (33.3%)	6 (12.2%)	0.058
LGE-CMR			
LVEF, %	35.6 ± 13.3	47.6 ± 14.8	0.008
LVEF ≤35%	7 (50.0%)	10 (20.4%)	0.028
LVEF>50%	1 (7.1%)	23 (46.9%)	0.007
LV mass (g)	153.85 (128.63, 174.1)	143.8 (112.8, 173.75)	0.710
RVEF (%)	50.3 ± 16.7	53.8 ± 9.8	0.408
grey zone%	14.0 (10.9, 14.7)	5.7 (2.3, 9.2)	<0.001
core scar%	10.0 (5.9, 14.8)	2.2 (1.1, 4.7)	<0.001
Simulation			
VT Inducibility	50 (22.9, 66.9)	0 (0, 21.1)	<0.001
VT circuits	2 (2, 4)	0 (0, 2)	<0.001
Integrated parameter			
LGE-VTsim index	69.0 (36.1, 86.5)	6.19 (3.0, 40.56)	<0.001

ACEI, angiotensin-converting enzyme inhibitor; ARB, angiotensin II receptor blocker; LGE, late gadolinium enhancement; LV, left ventricular; LVEF, left ventricular ejection fraction; NYHA, New York Heart Association; RVEF, right ventricular ejection fraction; VT, ventricular tachycardia. N/A: Not applicable; P values were derived from Fisher's exact test.

No significant differences in scar quantification or distribution were observed between patients with ICM (*n* = 45) and NICM (*n* = 20), likely reflecting heterogeneity within both groups. The distinction between ischaemic and non-ischaemic cardiomyopathy, together with their respective scar patterns, is illustrated in the Supplementary Materials.

The mean simulated VT induction rate, reflecting simulated electrophysiological propensity, was 5.6 (0.0, 37.5) %, with a mean of 1 (0, 2) inducible VTs per patient. To assess the clinical relevance of simulated sustained VT events, simulation outputs were compared with real-world electrophysiological and ablation findings. Among the 5 patients with VT who underwent EPS and catheter ablation, the real-world alignment with virtual VT circuits was as follows: In Patient 1, the clinical VT was non-inducible during EPS; however, the exit site identified by isochronal late activation mapping and pace mapping matched one of the virtual VT circuits. In Patients 2–4, each presented with a single monomorphic VT; the actual VT circuits correlated with the predicted virtual VT circuits, and ablation guided by these sites was successful. All 4 of these patients remained free of recurrent VT. In Patient 5, two VTs were induced during EPS, both of which aligned with 2 of the 5 virtual VT circuits identified. This patient experienced VT recurrence one week after ablation, with a morphology different from the previously documented VTs. This comparison suggests that virtual VT simulations can reflect actual arrhythmogenic substrates in patients with sustained VT and should be interpreted as probabilistic estimates of individualised risk.

### 3.3 Discriminatory performance of LVEF for sustained VT

In the overall population (*n* = 82), both LVEF ≤35% (*P* = 0.016) and lower LVEF as a continuous variable (*P* = 0.008) were significantly associated with sustained VT (Table 1). LVEF demonstrated moderate discriminatory performance, with a C-statistic of 0.730 (95% CI: 0.597–0.863). The conventional LVEF threshold of ≤35% showed limited sensitivity (50.0%) despite relatively high specificity (80.6%), whereas the Youden-derived optimal cut-off of 50.4% improved sensitivity (92.9%) at the expense of lower specificity (46.3%).

Among LGE-positive patients, those with sustained VT had significantly lower LVEF than those without sustained VT (35.6% ± 13.3% vs 45.4% ± 14.4%, *P* = 0.008), with a corresponding C-statistic of 0.730 (95% CI: 0.592–0.867).

### 3.4 Discriminatory value of visually assessed LGE for sustained VT

All patients with sustained VT were LGE-positive, whereas no VT events occurred in LGE-negative patients during the peri-CMR period, consistent with previous studies (19). Although visual LGE positivity differed significantly between patients with and without sustained VT (*P* = 0.023; Table 1), its discriminatory performance was modest (C-statistic: 0.634, 95% CI: 0.500–0.769) and did not differ significantly from that of LVEF (DeLong *P* = 0.200). These findings indicate that visual LGE assessment alone is insufficient for precise VT risk stratification and support the need for more advanced assessment among LGE-positive patients.

### 3.5 Development of an integrated LGE-VTsim Index

To construct an integrated discriminative model, scar and simulation parameters were compared between patients with and without sustained VT. The Event Group exhibited significantly larger core scar (10.0 (5.9, 14.8)% vs 2.2 (1.1, 4.7)%; *P* < 0.001 and grey zone [14.0 (10.9, 14.7)% vs 5.7 (2.3, 9.2)%; *P* < 0.001], higher virtual VT induction rates [50 (22.9, 66.9)% vs 0 (0, 21.1)%, *P* < 0.001], and a greater number of inducible VTs [2 (2, 4)vs. 0 (0, 2), *P* < 0.001].

Given the strong intercorrelations among these parameters (Spearman coefficients >0.7, Supplementary Table S2), EFA was performed to develop the LGE-VTsim index to reduce data dimensionality. The suitability of the data for factor analysis was confirmed by the Kaiser–Meyer–Olkin (KMO) measure (KMO = 0.742) and Bartlett's test (*P* < 0.001). LGE-VTsim index = 0.862 × Grey Zone + 0.614 × Core Scar + 0.906 × VT Inducibility + 0.913 × VT Circuits.

**Figure 2:**
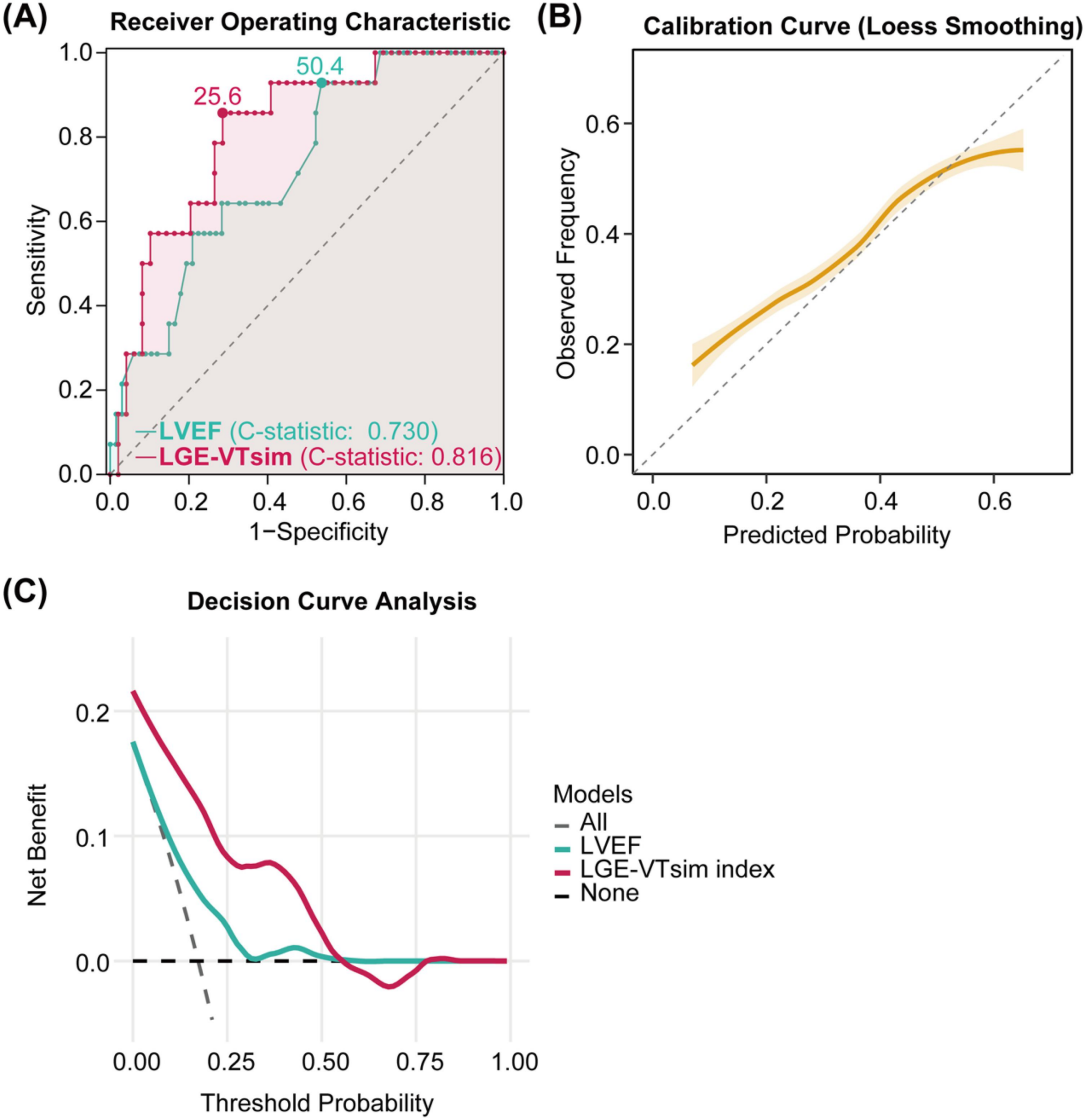
LGE–vTsim index for differentiating sustained VT events. (A) The ROC analysis of the novel LGE-VTsim index (pink, C-statistics = 0.816) and the conventional LVEF metric (teal curve, C-statistics = 0.730) in discriminating sustained VT events. Optimal cutoffs (determined by the Youden's index) are marked: 25.6 for LGE-VTsim and 50.4 for LVEF. **(B)** Calibration curve (with loess smoothing) of the LGE-VTsim index, illustrating the agreement between predicted probabilities of sustained VT and observed event frequencies. The dashed line represents perfect calibration (where predicted probability = observed frequency), and the shaded area denotes the 95% confidence interval reflecting estimation uncertainty. **(C)** Decision curve analysis comparing the net benefit of using the LGE-VTsim index (red line), LVEF (teal line), for predicting sustained VT, relative to a “None” strategy (horizontal black dashed line). The *x*-axis represents predicted probability thresholds, and the *y*-axis represents net benefit. ROC, receiver operating characteristic; LVEF, left ventricular ejection fraction; C-index, concordance index; LGE-VTsim index = 0.862× Grey Zone + 0.614 × Core Scar + 0.906× VT Inducibility + 0.913 × VT circuits.

Among LGE-positive patients, the LGE-VTsim index achieved a C-statistic of 0.816 (95% CI: 0.699–0.933) for identifying sustained VT, Of which, At a cut-off value >25.6, sensitivity was 85.7% (95% CI: 57.2%–98.2%) and specificity was 71.4% (95% CI: 56.7%–83.4%) (Figure 2A). The index remained independently associated with sustained VT after adjustment for LVEF (OR: 1.029, 95% CI: 1.008–1.051; *P* = 0.007), whereas LVEF lost statistical significance (OR: 0.952, 95% CI: 0.903–1.003; *P* = 0.064). Notably, when analysed as a dichotomous variable (LGE-VTsim >25.6 vs ≤25.6), logistic regression demonstrated a markedly stronger association (regression coefficient = 2.708, OR = 15.000, 95% CI: 2.968–75.810, *P* < 0.001). These findings indicate the incremental discriminative value of LGE-VTsim index beyond LVEF alone.

To assess the risk of overfitting, internal validation was performed using 1,000 bootstrap samples. The optimism-corrected calibration slope was 1.017, and the optimism-corrected C-statistic was 0.814 (original C-statistic: 0.816), indicating that the model maintained good discrimination without evidence of overfitting.

In addition, Locally Weighted Scatterplot Smoothing was applied to visualize the relationship between predicted probabilities and observed frequencies. The calibration plot (Figure 2B) demonstrated agreement between predicted and observed risks. The model achieved a Mean Absolute Error of 0.047 and a Mean Squared Error of 0.00366. An Mean Absolute Error below 0.05 indicates that, on average, predictions deviated from actual outcomes by less than 5%. The ideal calibration line remained within this interval across most of the risk spectrum, supporting the reliability of the model.

### 3.6 Incremental discriminative value of the LGE-VTsim Index over LVEF

Although the LGE-VTsim index demonstrated a numerically higher C-statistic compared with LVEF alone, DeLong's test (*P* = 0.352) did not reveal a statistically significant improvement, likely owing to the limited sample size. DCA (Figure 2C) **s**howed a trend toward increased net benefit with LGE-VTsim index across a range of threshold probabilities.

To further evaluate the incremental value, model comparison was performed between LVEF alone and a model incorporating both LVEF and the LGE-VTsim index. Adding the LGE-VTsim index improved overall discrimination; However, not all improvement metrics reached statistical significance. Model comparisons demonstrated a *Δ*C of 0.093 (95% CI: −0.199–0.014, *P* = 0.089) and an NRI of 70.0% (95% CI: 0.116–1.284, *P* = 0.019). A statistically significant IDI of 15.23% was also observed (95% CI: 0.039–0.266, *P* = 0.008).

### 3.7 Differences between sustained and non-sustained VAs

The ability of the LGE-VTsim index to discriminate between sustained and non-sustained VT was further evaluated among the 36 patients with documented VAs (21 non-sustained and 15 sustained). While neither visual LGE assessment nor LVEF significantly differentiated between the two groups, quantitative LGE parameters, simulation metrics, and the LGE-VTsim index all demonstrated significant discriminatory capacity (Table 3). These findings indicate that not all VAs reflect equivalent underlying substrate severity or susceptibility to sustained episodes, thereby supporting the clinical utility of the LGE-VTsim index for refined risk stratification in this population.

**Table 3:** Characteristics between sustained VT vs non-sustained VA.

Characteristic	Sustained VT (*n* = 15)	Non-sustained VA (*n* = 21)	P
Female (%)	1 (6.7%)	3 (14.3%)	0.626
Age (years)	56.0 (53.5, 68.5)	62.0 (53.0, 69.0)	0.835
Body surface area (m^2^)	1.91 ± 0.12	1.87 ± 0.15	0.398
Ventricular arrhythmia Burden %	0.3 (0.2, 0.95)	5 (2, 17.8)	< 0.001
Diabetes	4 (30.8%)	5 (23.8%)	0.655
Hypertension	5 (38.5%)	9 (42.9%)	0.800
Ischemic cardiomyopathy	9 (60.0%)	12 (57.1%)	0.864
NYHA function class			
I	1 (6.7%)	4 (19.0%)	0.858
II	8 (53.3%)	10 (47.6%)	
III	5 (33.3%)	6 (28.6%)	
IV	1 (6.7%)	1 (4.8%)	
Medications			
β-blocker	15 (100%)	18 (85.7%)	0.250
ACEI/ARB	15 (100%)	16 (76.2%)	0.062
Aldosterone antagonist	14 (93.3%)	12 (57.1%)	0.017
Loop diuretic	5 (33.3%)	4 (19.0%)	0.329
LGE-CMR			
LVEF, %	35.6 ± 13.3	43.9 ± 14.4	0.097
LVEF≤35% (%)	7 (46.7%)	5 (23.8%)	0.110
LVEF>50%	1 (6.7%)	7 (33.3%)	0.108
LV mass (g)	153.9 (128.6, 174.1)	147.3 (118, 184.4)	0.853
RVEF (%)	50.3 ± 16.7	51.7 ± 11.6	0.810
LGE-positive	15 (100%)	19 (90.5%)	0.500
grey zone %	14.0 (10.9, 14.7)	7.3 (3.1, 13.8)	0.018
core scar %	10.0 (5.9, 14.8)	3.0 (1.4, 5.9)	<0.001
Simulation			
VT inducibility	50.0 (22.9, 66.9)	12.5 (0.0, 31.3)	0.020
inducible VT circuits	2 (2, 4)	1 (0, 3)	0.039
Integrated parameter			
LGE-VT sim index	69.0 (36.1, 86.5)	20.0 (4.0, 50.3)	0.024

ACEI, angiotensin-converting enzyme inhibitor; ARB, angiotensin II receptor blocker; LGE, late gadolinium enhancement; LV, left ventricular; LVEF, left ventricular ejection fraction; NYHA, New York Heart Association; RVEF, right ventricular ejection fraction; VA, ventricular arrhythmia; VT, ventricular tachycardia. N/A: Not applicable; P values were derived from Fisher's exact test or independent samples t-test as appropriate.

## 4 Discussion

This study developed a novel LGE-VTsim index that integrates quantitative LGE-CMR scar characteristics with computational VT simulation parameters. The index demonstrated promising performance beyond LVEF for identifying sustained VT in Chinese patients with SHD. The LGE-VTsim index also effectively differentiated sustained VT from non-sustained VAs.

Current reliance on an LVEF ≤35% threshold to guide ICD therapy has well-documented limitations. Approximately 60%–70% of SCD events occur in patients with LVEF above this threshold (3, 20, 21), and many individuals with reduced LVEF may never derive benefit from ICD implantation. For example, in the conventional therapy group of the MADIT-II trial, the SCD rate was 10.0% (22, 23), which was higher than in the ICD group (3.8%), yet indicating that the vast majority of patients did not experience fatal arrhythmias. This observation highlights the limitations of a uniform LVEF-based threshold. Consistent with this, our results confirmed the suboptimal discriminatory performance of LVEF, with an optimal cut-off of ≤50.4%, suggesting that patients with higher values may be considered at low risk. The conventional LVEF ≤35% threshold was retained in LGE-negative individuals, in accordance with current guideline recommendations.

Invasive electrophysiological study (EPS) can aid risk stratification, particularly in patients with relatively preserved LVEF. The PRESERVE-EF study demonstrated that EPS effectively identified high-risk patients in a chronic ICM population with LVEF ≥40%, as malignant arrhythmias occurred exclusively in those with inducible VT (24). Although invasive EPS provides valuable prognostic information, non-invasive alternatives remain desirable.

In this context, LGE-CMR has emerged as a powerful tool for substrate-based risk assessment across various SHD aetiologies. In ICM, LGE-quantified infarct size independently predicts arrhythmic events (8), while in NICM, the presence of LGE is similarly associated with increased mortality and arrhythmic risk, as demonstrated in the DANISH-MRI sub-study (9). However, in our population, visual LGE assessment alone showed only modest discriminatory performance, and its C-statistic did not exceed that of LVEF. This finding underscores the limitations of qualitative evaluation and highlights the need for more individualized, quantitative scar analysis.

Computational VT simulation, based on patient-specific LGE-CMR–derived digital twin models, operates on principles analogous to invasive EPS but enables exhaustive pacing from multiple virtual sites to systematically assess arrhythmia susceptibility. This technique has been validated across diverse SHD populations, including HCM (25), cardiac sarcoidosis (26), tetralogy of Fallot (27), and post-infarction patients (27), with simulated VT inducibility consistently correlating with clinical outcomes. The present study extends these observations by integrating quantitative LGE characteristics (core scar, grey zone) with simulation-derived parameters (VT inducibility rate, number of circuits) into a unified index. The LGE-VTsim index demonstrated significantly improved discrimination for sustained VT compared with LVEF alone and remained independently associated with sustained VT after adjustment for LVEF.

Importantly, the LGE-VTsim index also demonstrated the ability to differentiate non-sustained VAs from sustained VT. Although the Improve SCA study identified frequent PVCs as a risk factor for SCD in primary prevention (28), our findings revealed significant differences in both LGE characteristics and in silico VT inducibility between these patient groups. Notably, the vast majority (13 out of 14) of patients with sustained VT showed no evidence of frequent PVCs on electrocardiographic monitoring. These results suggest that not all VAs confer equivalent clinical risk and highlight the potential of the LGE-VTsim index to improve risk stratification by identifying patients with a truly high-risk arrhythmogenic substrate.

**Figure 3:**
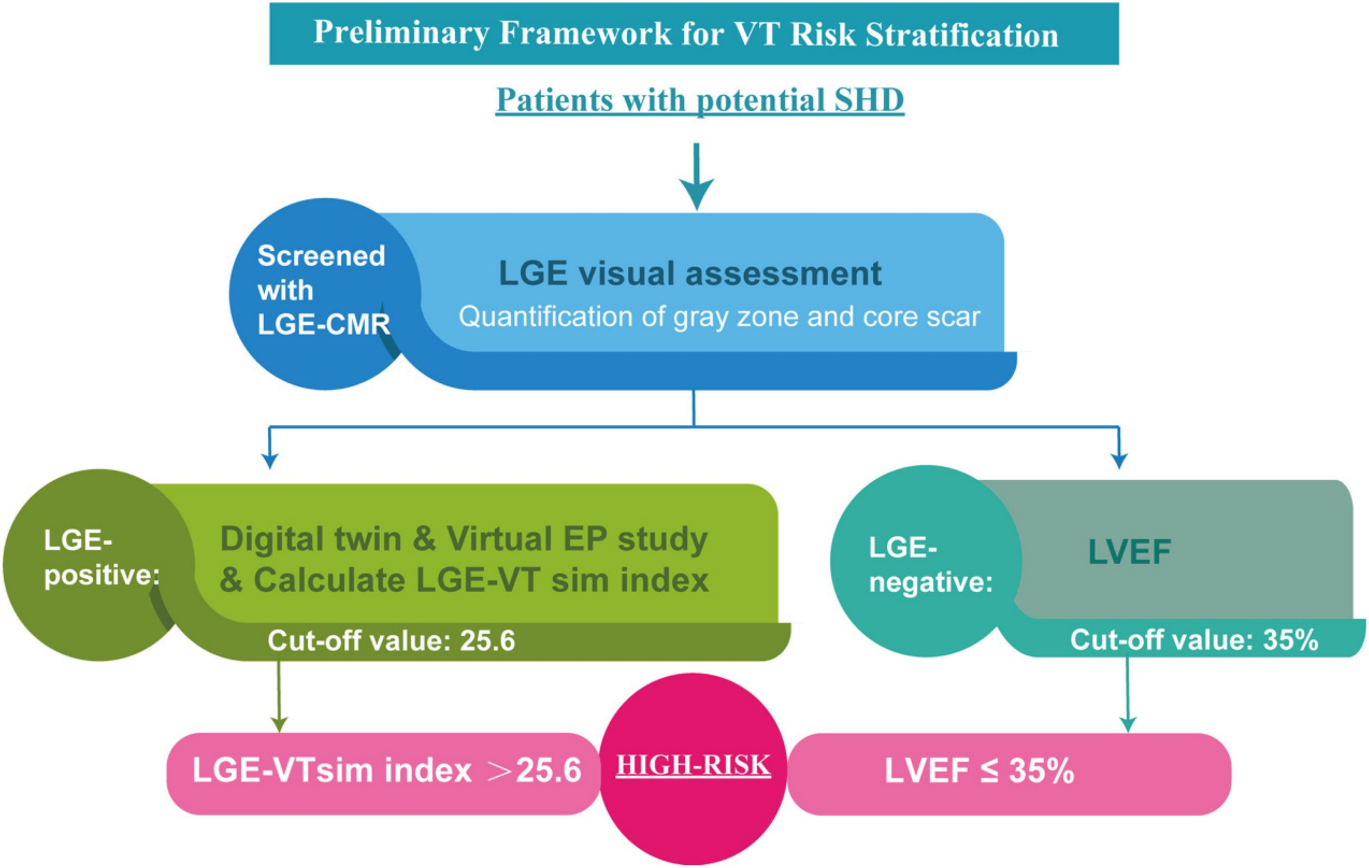
Sustained VT risk discrimination based on LGE-VTsim index. This flowchart presents an exploratory stepwise framework for VT risk stratification in suspected SHD. LGE-CMR screening first divides patients: LGE-positive cases undergo digital twin reconstruction and VT simulation to derive an LGE-VTsim index (>25.6 indicating high risk), while LGE-negative cases are stratified by LVEF (≤35 indicating high risk as conventionally suggested). This integrated approach enhances risk recognition beyond LVEF alone. CMR, cardiac magnetic resonance; LGE, late gadolinium enhancement; LVEF, left ventricular ejection fraction; SHD, structural heart disease; VT, ventricular tachycardia.

Based on the present findings, the LGE VTsim index represents a promising hypothesis-generating tool for sustained VT risk identification in patients with suspected SHD (Figure 3). Initial risk categorisation incorporated LVEF and LGE-VTsim index status: the combination of LGE-negative status and LVEF >50.4% identified patients at low risk, whereas the conventional LVEF ≤35% threshold served as a high-risk marker owing to its high specificity. Among LGE-positive patients, for whom visual assessment showed limited accuracy, the LGE-VTsim index effectively identified high-risk individuals and distinguished sustained from non-sustained VAs. At a cut-off value of >25.6, the index demonstrated high sensitivity and good specificity. What's more, advances in machine learning models leveraging ECG, imaging, and clinical data showed promises to improve arrhythmic risk prediction (29). However, corresponding regulatory frameworks are yet to be established for AI guided arrhythmic risk prediction.

**Figure 4:**
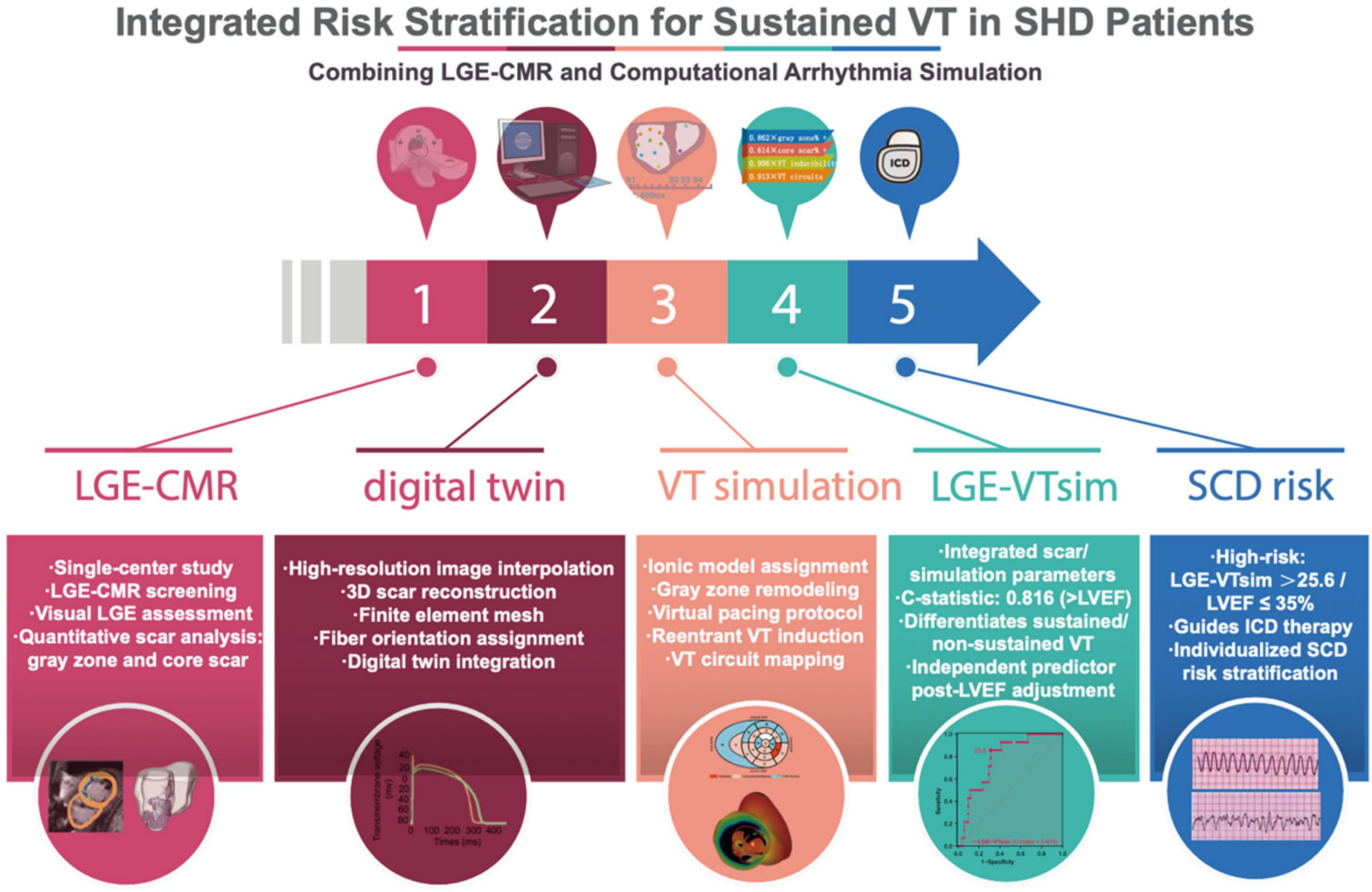
Central Illustration. This study developed a hypothesis-generating parameter, named LGE-VTsim index, incorporating LGE-CMR for scar quantification, and virtual VT inducibility and circuits. the proposed method overcomes limitations of traditional LVEF-based risk assessment. LGE-CMR, late gadolinium enhancement cardiac magnetic resonance; VT, ventricular tachycardia; SCD, sudden cardiac death; SHD, structural heart disease; LVEF, left ventricular ejection fraction.

To our knowledge, this is the first study to explore the feasibility of combining LGE-CMR with computational simulation in a Chinese population. The exploratory framework employs LGE-CMR for initial screening and applies the LGE-VTsim index for advanced assessment in LGE-positive patients, providing a pathophysiology-informed and individualised approach to VT assessment (Figure 4). This parameter appears particularly valuable for optimising risk evaluation in patients with LVEF between 35% and 50.4%, a population for whom current clinical guidelines offer limited specific guidance.

## 5 Limitations

Several limitations should be acknowledged. First, the single-centre design and limited sample size increase the risk of overfitting, necessitating external validation in larger, multicentre aetiology-stratified cohort. Future studies should incorporate inter-observer reproducibility and compare findings with invasive electrophysiological study to confirm physiological relevance. Second, the 6-month arrhythmia window and a single baseline LGE-CMR examination may capture disease progression; serial CMR with updated LGE-VTsim index calculations longitudinal risk evaluation. Third, the cohort was predominantly male (83.1%), potentially limiting generalisability to women. Sex-based differences in myocardial scarring—with women exhibiting less extensive fibrosis—may contribute to lower malignant arrhythmia rates and underscore the need for sex-sensitive risk stratification beyond LVEF (30, 31), pending future HCM specific validation (32), Finally, although *β*-blocker therapy was not adjusted for in the model, its widespread use among patients with sustained VT supports the model's applicability to treated populations.

## 6 Conclusion

This study demonstrates the feasibility of combining LGE-CMR with computational VT simulation in a Chinese population and proposes the LGE-VTsim index as a hypothesis-generating parameter for identifying sustained VT in SHD. Compared with LVEF alone, the LGE-VTsim index showed improved trend of discriminative performance over LVEF alone and effectively distinguished sustained from non-sustained arrhythmias.

## Data Availability

The raw data supporting the conclusions of this article will be made available by the authors, without undue reservation.
