# Supplementary material for: Discrimination of sustained VT in structural heart disease using LGE-CMR and computational simulation: moving beyond LVEF
Source: Front Cardiovasc Med. 2026 Jul 7;13:1870223. doi: 10.3389/fcvm.2026.1870223 (PMC13385657; doi:10.3389/fcvm.2026.1870223)
Supplement: Supplementary file 1 [file Datasheet1.docx]

**SUPPLEMENTARY MATERIALS**

**1. Detailed methodological descriptions about image acquisition, digital twin reconstruction, and electrophysiological simulation**

***LGE-CMR Image Acquisition and Analysis***

All participants underwent LGE-CMR examination on 3-T scanners (Siemens Prisma or Vida). Long- and short-axis cine images were acquired using electrocardiographic gating and breath-hold steady-state free precession sequences. LGE imaging was performed 10-15 minutes after intravenous administration of contrast (0.1 mmol/kg gadopentetate dimeglumine; BeiLu Pharmaceutical, Beijing, China) using an inversion-recovery gradient-echo sequence aligned with cine orientations. Key sequence parameters included a slice thickness of 8 mm, a gap of 2 mm, and an in-plane resolution of 1.6×1.6 mm; the inversion time was optimized to null normal myocardium. Specific LGE sequence parameters were as follows: echo time/repetition time = 1.96/450 ms, flip angle = 20°.

For each LGE-CMR slice, epicardial and endocardial contours were initially obtained using an automatic segmentation algorithm[1]. These contours were subsequently reviewed and manually refined by two experienced observers to ensure accurate delineation of myocardial borders, with careful exclusion of blood pools and epicardial fat. Myocardial tissue was classified into non-scar and scar regions using a Gaussian mixture model-based method, as detailed in our prior publication[2]. The scar region was further subdivided into gray zone and core scar components according to our previously established methodology[3]: after determining the maximum and minimum pixel intensities within the scar region, pixels with intensities greater than 50% of the intensity range (intensity_max – intensity_min) were classified as core scar, and the remaining pixels were designated as gray zone.

***Heart Digital Twin Reconstruction***

Following image segmentation, the CardioViz3D (INRIA, Sophia Antipolis, France) was used to interpolate the segmented low-resolution images to a higher spatial resolution, resulting in a voxel size of approximately 0.4 mm. The three-dimensional (3D) geometry of the infarcted tissue—comprising both gray zone and the core scar —— was reconstructed using the log-odds method[4] and subsequently intergrated with the corresponding high-resolution ventricular images.

A finite element mesh was generated from each patient-specific biventricular geometry using Mimics (Materialise NV, Leuven, Belgium). As reported in our previous work[5], the average element size in these meshes was approximately 400 μm. Myocardial fiber orientation was assigned using a validated rule-based method[6], which utilizes a Laplace-Dirichlet algorithm to define local transmural and apicobasal directions, followed by bidirectional spherical linear interpolation to assign fiber orientations ranging from −40°at the epicardium to +65°at the endocardium[7, 8].

***Electrophysiological modeling***

The electrophysiological properties of non-infarcted myocardium was represented using the ten Tusscher ventricular myocyte model[9]. To simulate the electrophysiological remodeling in the gray zone, the conductances of key ionic currents were scaled based on previously published experimental data: the fast delayed rectifier potassium current (IKr) was reduced to 30% of its normal value, the slow delayed rectifier potassium current (IKs) to 20%, the L-type calcium current (ICaL) to 31%, and the sodium current (INa) to 38% [10, 11]. Conductivity parameters were set to achieve conduction velocities of approximately 0.60 m/s in non-infarct myocardium and 0.30 m/s in the gray zone, consistent with clinically reported values[12]. The core scar was treated as electrically passive tissue.

***VA Induction in Digital Twin***

VT inducibility and reentrant circuit mapping were simulated by numerically solving the reaction–diffusion equations using the openCARP electrophysiology environment[13]. Simulations were performed on high-performance computing clusters at Dalian University of Technology, China.

Ventricular pacing sites were selected according to the American Heart Association (AHA) segmentation: six sites in the left ventricle adjacent to the infarct border zone, and two additional sites in the right ventricle (outflow tract and apex). A standardized virtual rapid pacing protocol, adapted from our previous work, was applied at each site to assess VT inducibility. The protocol consisted of a 6-beat S1 train (600 ms) followed by up to three progressively premature stimuli (S2-S4) with progressively shorter intervals[14].

If a reentrant VT was induced, the arrhythmia was simulated for 10 seconds to confirm its stability. The resulting VT circuits and the anatomical locations of the reentrant circuits at the end of the 10-second simulation were recorded for analysis.

**References:**

1 Fu Z, Zhang J, Luo R, et al. TF-Unet:An automatic cardiac MRI image segmentation method. *Math Biosci Eng* 2022;**19**:5207-22.

2 Wu ZH, Sun LP, Liu YL, et al. Fully Automatic Scar Segmentation for Late Gadolinium Enhancement MRI Images in Left Ventricle with Myocardial Infarction. *Curr Med Sci* 2021;**41**:398-404.

3 Tong L, Zhao C, Fu Z, et al. Preliminary Study: Learning the Impact of Simulation Time on Reentry Location and Morphology Induced by Personalized Cardiac Modeling. *Front Physiol* 2021;**12**:733500.

4 Ukwatta E, Arevalo H, Rajchl M, et al. Image-based reconstruction of three-dimensional myocardial infarct geometry for patient-specific modeling of cardiac electrophysiology. *Med Phys* 2015;**42**:4579-90.

5 Cao B, Zhang N, Fu Z, et al. Studying the Influence of Finite Element Mesh Size on the Accuracy of Ventricular Tachycardia Simulation. *Rev Cardiovasc Med* 2023;**24**:351.

6 Bayer JD, Blake RC, Plank G, et al. A novel rule-based algorithm for assigning myocardial fiber orientation to computational heart models. *Ann Biomed Eng* 2012;**40**:2243-54.

7 Lombaert H, Peyrat JM, Croisille P, et al. Human atlas of the cardiac fiber architecture: study on a healthy population. *IEEE Trans Med Imaging* 2012;**31**:1436-47.

8 Eggen MD, Swingen CM, Iaizzo PA. Ex vivo diffusion tensor MRI of human hearts: relative effects of specimen decomposition. *Magn Reson Med* 2012;**67**:1703-9.

9 ten Tusscher KH, Panfilov AV. Alternans and spiral breakup in a human ventricular tissue model. *Am J Physiol Heart Circ Physiol* 2006;**291**:H1088-100.

10 Deng D, Arevalo HJ, Prakosa A, et al. A feasibility study of arrhythmia risk prediction in patients with myocardial infarction and preserved ejection fraction. *Europace* 2016;**18**:iv60-iv6.

11 Arevalo H, Plank G, Helm P, et al. Tachycardia in post-infarction hearts: insights from 3D image-based ventricular models. *PLoS One* 2013;**8**:e68872.

12 Anter E, Tschabrunn CM, Buxton AE, et al. High-Resolution Mapping of Postinfarction Reentrant Ventricular Tachycardia: Electrophysiological Characterization of the Circuit. *Circulation* 2016;**134**:314-27.

13 Plank G, Loewe A, Neic A, et al. The openCARP simulation environment for cardiac electrophysiology. *Comput Methods Programs Biomed* 2021;**208**:106223.

14 Dong R, Fu Z, Zhang C, et al. Comparative analysis of the ten Tusscher and Tomek human ventricular cell models at cellular, tissue, and organ levels: Implications for post-infarct ventricular tachycardia simulation. *Physiol Rep* 2025;**13**:e70435.

**2. Differences in scar patterns between ischemic and non-ischemic cardiomyopathy**

Existing evidence indicates differences in the benefit of primary prevention ICD therapy between ICM and NICM patients (Witt et al., Europace, 2016), suggesting that LVEF alone may be insufficient for accurate arrhythmic risk prediction in NICM. However, meta-analyses have confirmed that LGE on CMR imaging effectively predicts SCD risk in both ICM and NICM (Disertori et al., JACC: Cardiovascular Imaging, 2016). So we further explore whether the CMR-derived scar patterns were different between ICM and NICM.
 Among the 82 patients included, 53 were diagnosed with ICM and 29 with NICM. Detailed characteristics of the study population are presented in Table S1. Patients with NICM tended to be younger. There were no statistically significant differences in scar burden, simulated VA, or real-world VA events between the two groups.
 In our cohort, no significant differences in scar quantification or distribution were observed between ICM and NICM patients, likely due to heterogeneity within both groups. Among ICM patients, infarct-related arteries included the left anterior descending (LAD, n=17), right coronary artery (RCA, n=15), and multi-vessel disease (n=21), with specific involvement as follows: 7 cases involved LAD and RCA, 2 involved RCA and left circumflex artery (LCX), 1 involved LAD and LCX, and 11 involved all three vessels. Among NICM patients, included dilated cardiomyopathy (n=16), hypertrophic cardiomyopathy (n=9), and other types (n=4, comprising 2 cases of sarcoidosis and 2 cases of amyloidosis), the distribution of etiologies may vary across different regions.
 And the detailed analysis of scar distribution revealed the following patterns (Fig. S1). Anterior wall scars in ICM were primarily associated with LAD lesions, though scar formation distal to the occlusion led to a slightly lower prevalence near the valve annulus. In NICM, anterior scars were predominantly linked to hypertrophic cardiomyopathy, and the proportion of HCM cases aligned with the observed scar distribution.
 Septal scars in ICM commonly resulted from combined LAD and RCA involvement, which was frequent in our cohort. In NICM, septal scars were associated with hypertrophic cardiomyopathy, dilated cardiomyopathy, and sarcoidosis, collectively accounting for the majority of cases.
 Inferior wall scars in ICM were predominantly due to RCA occlusion (consistent with right coronary dominance in the Chinese population), making them the second most common type. In NICM, inferior scars were mainly attributed to hypertrophic cardiomyopathy and cardiac sarcoidosis. The etiological distribution aligns with the proportion of inferior wall scars in NICM.
 Apical scars were relatively uncommon in NICM and were primarily observed in HCM patients with apical hypertrophy and ventricular aneurysm formation. In ICM, apical scars were related to LAD lesions or RCA distal branch involvement, though their prevalence was mitigated by timely revascularization and collateral circulation.
 Lateral wall scars were the least common in both groups. In ICM, they were associated with LCX involvement, which was infrequent in our cohort. In NICM, lateral scars occurred primarily in patients with sarcoidosis or post-myocarditis cardiomyopathy, both of which were underrepresented.
 In summary, while scar distribution exhibits etiology-specific patterns in individual cases or specific diseases, the overall regional scar distribution did not differ significantly between ICM and NICM in our cohort, likely due to the heterogeneity of vascular involvement in ICM and the diversity of etiologies in NICM. We have acknowledged this limitation and emphasize the need for future studies with larger, etiology-stratified cohorts to further elucidate subtype-specific risk patterns.

**Table S1. Baseline Characteristics of the ICM and NICM patients**

|  | ICM (n=53) | NICM (n=29) | P |
| --- | --- | --- | --- |
| Female | 7 (13.2%) | 7 (24.1%) | 0.209 |
| Age (years) | 62 (56,69) | 55 (43, 64) | 0.013 |
| Body surface area (m^2^) | 1.86 ± 0.14 | 1.89 ± 0.23 | 0.378 |
| Ventricular arrhythmia Burden % | 0.3 (0.2,0.7) | 0.2 (0.2,3) | 0.259 |
| Diabetes | 16 (30.8%) | 8 (28.6%) | 0.838 |
| Hypertension | 28 (53.8%) | 14 (50%) | 0.742 |
| NYHA function class |  |  |  |
| I | 12 (22.6%) | 3 (10.3%) | 0.112 |
| II | 30 (56.6%) | 13 (44.8%) |  |
| III | 10 (18.9%) | 11 (37.9%) |  |
| IV | 1 (1.9%) | 2 (6.9%) |  |
| Medications |  |  |  |
| β-blocker | 44 (83%) | 22 (75.9%) | 0.434 |
| ACEI/ARB | 43 (81.1%) | 21 (72.4%) | 0.362 |
| Aldosterone antagonist | 19 (35.8%) | 19 (65.5%) | 0.010 |
| Loop diuretic | 9 (17%) | 7 (24.1%) | 0.434 |
| LGE-CMR |  |  |  |
| LVEF, % | 46.0±14.2 | 44.2 ±14.9 | 0.602 |
| LVEF ≤35% | 11 (21.2%) | 9 (31%) | 0.323 |
| LGE positive | 44 (83%) | 20 (69%) | 0.142 |
| LV mass (g) | 138.28 (106.32, 157.67) | 147.9 (121.95, 195.52) | 0.191 |
| RVEF (%) | 53.2 ±9.9 | 50.6 ±15.0 | 0.469 |
| gray zone% | 7.54 (2.545, 13.145) | 6.76 (4.38, 14.19) | 0.739 |
| core scar% | 4.32 (1.915, 9.62) | 1.635 (1.05, 4.6325) | 0.023 |
| Simulation |  |  |  |
| VT inducibility | 12.5 (0, 37.5) | 0 (0, 25) | 0.363 |
| VT circuits | 1 (0, 2) | 0 (0, 2) | 0.262 |
| Events |  |  |  |
| All VA | 21 (39.6%) | 15 (51.7%) | 0.291 |
| Sustained VT | 9 (17%) | 6 (20.7%) | 0.678 |

**Abbreviations**: ACEI, angiotensin-converting enzyme inhibitor; ARB, angiotensin II receptor blocker; LGE, late gadolinium enhancement; LV, left ventricular; LVEF, left ventricular ejection fraction; NYHA, New York Heart Association; RVEF, right ventricular ejection fraction; VA，Ventricular arrhythmia; VT, ventricular tachycardia. N/A: Not applicable; P values were derived from Fisher's exact test.

**Figure S1. Detailed analysis of scar distribution patterns between ICM and NICM.**

**
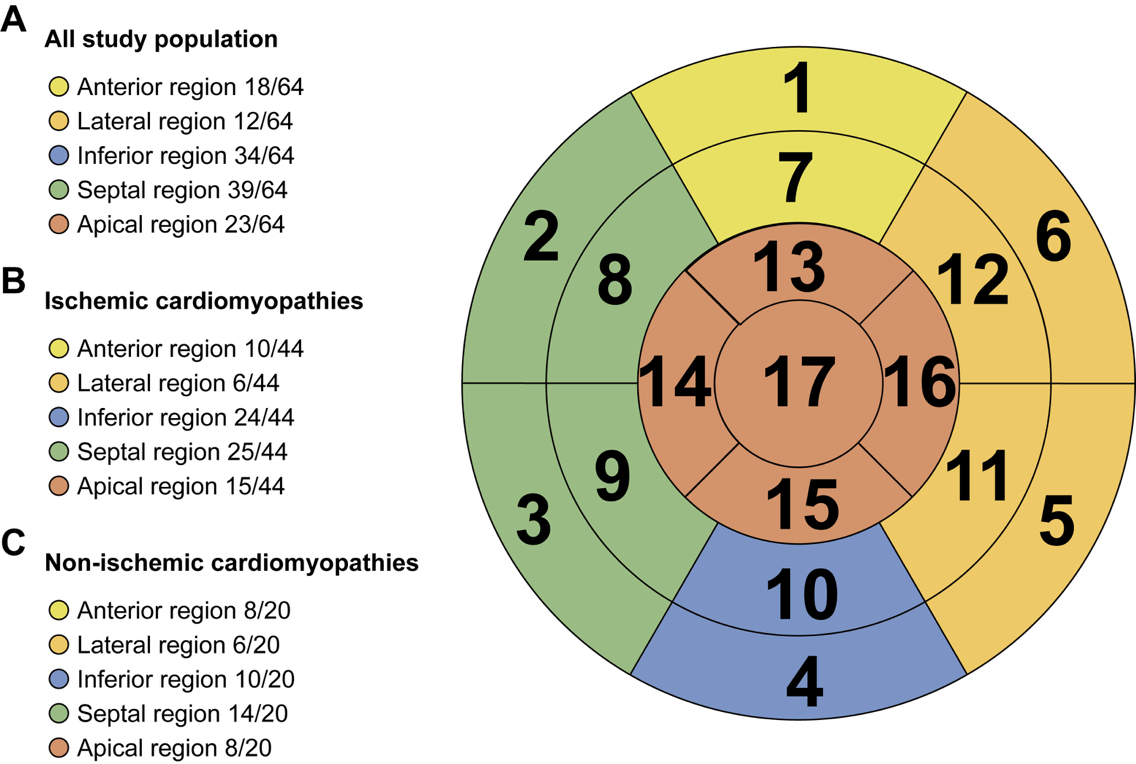
**

**3.**

**Table S2. Correlations among scar parameters, and VT simulation indices.**

|  |  | gray zone | core scar | VT inducibility | VT circuits |
| --- | --- | --- | --- | --- | --- |
| gray zone | rho | - | 0.716*** | 0.791*** | 0.799*** |
|  | P | - | <0.001 | <0.001 | <0.001 |
| core scar | rho | 0.716*** | - | 0.800*** | 0.785*** |
|  | P | <0.001 | - | <0.001 | <0.001 |
| VT inducibility | rho | 0.791*** | 0.800*** | - | 0.957*** |
|  | P | <0.001 | <0.001 | - | <0.001 |
| VT circuits | rho | 0.799*** | 0.785*** | 0.957*** | - |
|  | P | <0.001 | <0.001 | <0.001 | - |

**Abbreviations**: LVEF, left ventricular ejection fraction; VT, ventricular tachycardia; rho, Spearman's rank correlation coefficient;***p < 0.001 (two-tailed).
